# Leveraging Comprehensive Cancer Registry Data to Enable a Broad Range of Research, Audit and Patient Support Activities

**DOI:** 10.3390/cancers14174131

**Published:** 2022-08-26

**Authors:** Belinda Lee, Lucy Gately, Sheau Wen Lok, Ben Tran, Margaret Lee, Rachel Wong, Ben Markman, Kate Dunn, Vanessa Wong, Matthew Loft, Azim Jalili, Angelyn Anton, Richard To, Miles Andrews, Peter Gibbs

**Affiliations:** 1 Walter & Eliza Hall Institute of Medical Research, Parkville, VIC 3052, Australia; 2 Department of Medical Oncology, Northern Health, Epping, VIC 3076, Australia; 3 Department of Medical Oncology, Peter MacCallum Cancer Centre, Melbourne, VIC 3000, Australia; 4 School of Medicine and Dentistry, University of Melbourne, Parkville, VIC 3010, Australia; 5 Cabrini Haematology and Oncology Centre, Malvern, VIC 3144, Australia; 6 Department of Medical Oncology, Eastern Health, Melbourne, VIC 3151, Australia; 7 Department of Medical Oncology, Western Hospital, Melbourne, VIC 3021, Australia; 8 Eastern Health Clinical School, Monash University, Clayton, VIC 3800, Australia; 9 Department of Medical Oncology, Alfred Health, Melbourne, VIC 3004, Australia; 10 Department of Medical Oncology, Ballarat Health Service, Ballarat Central, VIC 3350, Australia

**Keywords:** cancer registry, registry-based trials, data-driven research, big data, digital health

## Abstract

**Simple Summary:**

Registry data has the potential to support a broad range of research, audit and education initiatives. Here, we describe the experience and learnings of a series of large multi-institutional cancer registries that leverage real-world clinical data for a range of purposes, that informs the conduct and output of each registry in a virtuous cycle. Lessons learnt include the need for careful and continuous curation of information being collected, regular database updates, and the need for a continued focus on data quality. As a standalone resource, each registry has supported numerous projects, but linkage with external datasets with patients in common has enhanced the research potential. Multiple projects have linked registry data with matched tissue specimens to support the discovery and valiation of prognostic and predictive markers in the tumour and blood specimens. Registry-based biomarker trials have been successfully supported, generating novel and practice-changing data. Registry-based clinical trials, particularly studies exploring the best use of drug options are now complementing the research conducted in traditional clinical trials. More recent projects supported by the registries include health economic studies, personalised patient education material, and increased consumer engagement, including consumer entered data.

**Abstract:**

Traditional cancer registries have often been siloed efforts, established by single groups with limited objectives. There is the potential for registry data to support a broad range of research, audit and education initiatives. Here, we describe the establishment of a series of comprehensive cancer registries across the spectrum of common solid cancers. The experience and learnings of each registry team as they develop, implement and then use collected data for a range of purposes, that informs the conduct and output of other registries in a virtuous cycle. Each registry is multi-site, multi-disciplinary and aims to collect data of maximal interest and value to a broad range of enquiry, which would be accessible to any researcher with a high-quality proposal. Lessons learnt include the need for careful and continuous curation of data fields, with regular database updates, and the need for a continued focus on data quality. The registry data as a standalone resource has supported numerous projects, but linkage with external datasets with patients in common has enhanced the audit and research potential. Multiple projects have linked registry data with matched tissue specimens to support prognostic and predictive biomarker studies, both validation and discovery. Registry-based biomarker trials have been successfully supported, generating novel and practice-changing data. Registry-based clinical trials, particularly randomised studies exploring the optimal use of available therapy options are now complementing the research conducted in traditional clinical trials. More recent projects supported by the registries include health economic studies, personalised patient education material, and increased consumer engagement, including consumer entered data.

## 1. Introduction

Much has been written about the potential of big data to transform medical research. The rapidly expanding volume and breadth of data captured for each patient, and the variety of databases in which this is captured, is ever expanding. This data, alongside data generated from newer “omic” technologies, is enabling increasingly varied and sophisticated analyses. However, to utilise the data to its fullest potential, the data quality must be prioritised, and the potential impact of known and unknown confounders duly considered, to avoid potentially erroneous or harmful conclusions.

Capturing high quality data necessarily goes beyond a review of the hospital medical record which does not capture patient and tumour detail, management and outcomes comprehensively. This issue, well described in the era of paper records [1], continues to pose a challenge in the electronic health record (EHR) era [2], in part because clinical software was designed primarily with administrative purposes in mind, rather than to support audit and research. For patients with cancer, important clinical details such as performance status, comorbidity, and key pathological features are frequently poorly documented, even though these often determine treatment choice and outcomes of interest.

Until recently, cancers were broadly characterized by anatomical site, pathology, and stage. Now however, the heterogeneity of cancer is increasingly understood, driving the interest in personalized medicine. Clinical registries represent an important opportunity to further refine our knowledge of how best to treat every individual patient, but large patient numbers and multi-site engagement are critical to support this effort. A major challenge is that many registry efforts remain siloed, often initiated by a single craft group (e.g., surgeons), to collect data from a limited time period (e.g., perioperative), with a broad single focus (e.g., audit), and limited multi-site engagement—all of which limit potential impact. National registries like the Surveillance, Epidemiology, and End Results (SEER) database in the United States or state-level registries are the gold standard, as authoritative sources for cancer statistics, but still only offer a core minimum dataset. Limitations to the analyses of data extracted from these state-level registries include the completeness of variables, biases associated with unmeasured rationale for treatment choices or whether treatment was not received or just not recorded, and lack of active follow-up of patients with granular level data on types of treatment received to enable measures of sequence outcomes.

**Figure 1:**
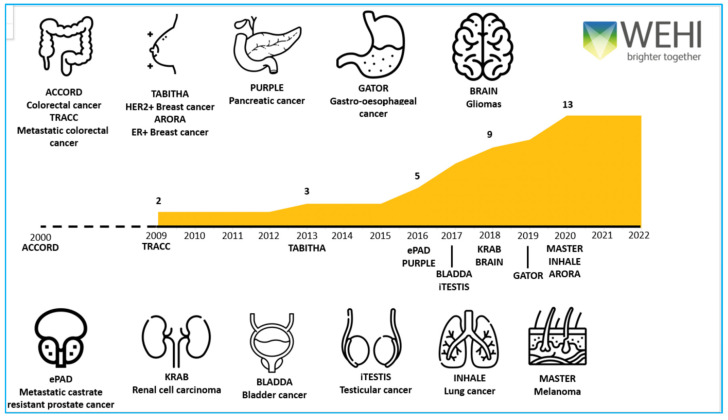
Solid tumour registries by date of protocol development.

Here, we discuss the lessons learnt in establishing the following multi-disciplinary, multi-site national and international cancer registries—ACCORD (colorectal cancer) TRACC (advanced colorectal cancer), TABITHA (HER2 breast cancer), AURORA (ER breast cancer), PURPLE (pancreatic cancer), GATOR (gastro-oesophageal cancer), BRAIN (gliomas), ePAD (prostate cancer), KRAB (kidney cancer), BLADDA (bladder cancer), iTESTIS (testicular cancer), INHALE (lung cancer) and MASTER (melanoma) which partner with 82 cancer centres across Australia, New Zealand, Hong Kong and Singapore Figure 1. We outline (1) the importance and logistics of multi-site and multi-disciplinary engagement; (2) the time and resources required to collect valuable longitudinal data; (3) our continued focus on data quality; and (4) the broad range of activities our registries support. Importantly, we highlight the value proposition of using the same datasets to support audit, research and education projects, and the data value framework required for data transformation. Combining registry data with external data sets, tumour tissue and/or blood-based analyses ensures maximum opportunity for impactful output, creates research efficiencies and focuses on sustainability.

## 2. Multi-Disciplinary and Multi-Site Engagement, with Robust Governance

Cancer care is increasingly complex and multi-disciplinary. Collecting data at a single time period, or from a single discipline, only provides a snapshot of a patient’s journey and limits audit and research opportunities. An inclusive multi-disciplinary approach supports engagement of all relevant craft groups to data collection and curation of datasets. It also creates a shared sense of data ownership, a broader range of potential research questions, and involvement in publications with appropriate acknowledgement in the form of co-authorship.

In the era of biomarker-defined patient subsets, each having distinct behaviour and increasingly distinct standards of care, multi-site registries provide the ability to pool data on rare patient populations. For example, for common tumours such as colorectal cancer where there is an ever-expanding list of clinically relevant biomarkers (e.g., *BRAF* V600E, MMR, *HER2*, *POLE*, *KRAS* G12C, *NTRK*), each patient subset represents only a small proportion of the overall population. Even large clinical sites may only manage a handful of such patients per year, multi-site participation can provide sufficient sample sizes for statistically meaningful analysis and is hence an essential component to maximise any registry’s impact. Multi-site registries also enable tracking of individual patients throughout the health system (e.g., second opinions, clinical trial opportunities) and comparison of practices and outcomes between institutions, which may have differing patient populations and treatment practices.

Historically, beyond state-lead registries, more granular level cancer data sets have been isolated within institutions, with limited collaboration. To address ownership issues, our policy is that each hospital has a data custodian who must authorise the use of any de-identified data contributed by that site to any combined analysis. Data security and privacy is maintained through a data tenancy framework that locks data access to each originating site. All sites are encouraged to actively participate in submitting data related projects. Research proposals may be submitted by any researcher, including those not associated with the registry, with all proposals including a clearly defined research question, and data management plan which is reviewed through an established quality and ethics framework. Each proposal must pass review by the relevant Registry Steering Committee, followed by a scientific and ethics review board, before being sent to each individual site’s data custodian for site approval and data release. All projects must adhere to standard guidelines about data use. This process and concept of data control has resulted in hundreds of approved projects, more than 100 publications from the colorectal databases alone, and sustained and enhanced site engagement. Additionally, the quality of research projects is improved, through the use of larger comprehensive multi-site data sets, more varied populations, and from the invaluable input offered by the Registry Steering Committee data owners, most of whom are experts in their chosen tumour stream.

The collection of robust multi-site data has also paved the way for international collaborations. The International Metastatic Database Consortium (IMDC) incorporates data from participating institutions in over 15 countries including contributions from our national KRAB database, generating findings that inform global treatment practices for kidney cancer patients.

## 3. Measures for Data Quality

Ultimately, the greatest challenge for any data collection effort is maintaining a high standard of data quality. Incomplete or inaccurate data is a particular risk when there is limited data monitoring or maintenance, or when potentially important confounders are not considered, compromising the quality and impact of research output. Deciding what not to include is just as important as what is included. A tendency to capture every potential data point can become onerous and override the willingness to contribute data. Relevant data fields need to be curated to ensure only those of high importance and reliability are captured. An effort to collect performance status and co-morbidity, whilst challenging, is worthwhile given the critical impact on treatment and outcomes. Similarly, short-term surgical outcomes (such as return to theatre rates) or serious adverse events (requiring hospitalisation), that are usually well documented, are important and readily captured. To ensure data accuracy, capturing data as close to real-time as possible is important. Oncology is an ever-evolving field, hence continuously reviewed data fields and adaptability of the registry is imperative to ensure data remains relevant.

An initial investment in a high-quality registry database can markedly enhance long-term usability and sustainability. The database should be visually attractive, intuitive to use, and limit the number of data sections to be filled for each patient. Some data only becomes relevant in the context of a specific patient subset, so the potential to hide and reveal questions as required creates efficiency. Inbuilt data quality checks include the use of a select number of mandatory critical data items to reduce missing data (i.e., data fields must be completed for every patient in order to submit the database form), dropdown data fields to ensure consistency of data entry, logic rules to reduce key stroke and other errors such as ranges for all values, accuracy rules (e.g., rectal cancer surgery options do not include a right hemicolectomy) and time-based rules (e.g., date of diagnosis cannot be after date of death).

To ensure completeness of variables each registries’ data architecture has been designed by clinical experts in the field, with an in-depth appreciation of key data points required to capture the entire patient cancer journey. This process is critical to building a comprehensive database that collates the data inputs required to enable correlative analyses and predictive modelling. Granular level data collated includes patient “performance status”, “co-morbidities”, “treatment intent”, “start and stop dates” of treatments, as well as specifics of surgical procedures. All data can be directly entered by the treating teams prospectively which also improves accuracy and timeliness of data entry.

Frequent use of the data for research and audit purposes ensure the quality of data is continually maintained. For some data fields, such as medicine related adverse event data, survival data or service utilisation data, the data field may be eminently important, but fiendishly difficult to collect in practice. Having an alternate data set that includes these fields allows a ready opportunity for audit and to complete missing fields. As such, data linkages become increasingly valuable. Table 1 outlines the data linkages currently in place for our registries and the value and data quality checks that they provide.

## 4. Support for a Range of Activities

Large cohorts of well curated, high quality, multi-site data provide an opportunity for a wide range of activities. Research proposals may be submitted by any of the participating centres, with statistical and bioinformatic support provided upon request. This improves research efficiency, return on the time and effort invested in establishing the clinical registry and adds value to the registry. A selection of examples are described below.

## 5. Expanded Data Linkage

Ethically approved linkages to alternate data sets that capture data on patients already included in a clinical registry offer opportunities to audit, validate and enrich data in the registry. All linkages with external datasets are made via a number of secure algorithms that ensure de-identification of all data and the use of unique linkage codes, with examples shown in Table 1. Increasingly electronic health records (EHR) are becoming more commonplace as hospitals and private practices move away from paper-based systems thus creating greater opportunities to set up automated data linkages between structured data from the EHR, administrative datasets and the registries. In addition, pharmacy databases capturing inpatient medications and radiation oncology databases can be used to capture or audit radiation therapy administered. Hospital administration databases capture additional information, such as patients’ preferred language, which allows analysis of the impact of language on cancer diagnosis and treatment [4,6,7,8]. Familial cancer screening databases provide an opportunity to explore referral for genetic counselling and subsequent results [3].

Crosschecking with local cancer registries can ensure consecutive cases diagnosed at each study site are identified and captured [5]. External governmental datasets can provide access to information on non-cancer prescriptions and procedures and improve the accuracy of survival data, which can be challenging for registries to maintain as many cancer patients die outside of the immediate hospital network.

### 5.1. Measuring Quality of Care

Delivering high quality patient care is the ultimate aim for healthcare, but defining it, and then measuring and reporting these quality metrics to create a virtuous cycle, often proves challenging. The utility of large administrative datasets may be compromised by the slow cadence of data reporting, the lack of data granularity and diminishing clinical relevance, as historical data rapidly becomes irrelevant as practice standards evolve. The utility of quality indicators first developed decades ago also needs to be continually reviewed [9]. Datasets should be as current as possible, incorporating relevant biomarkers, modern disease treatment modalities and current outcome standards, with careful selection of quality metrics. This is where a comprehensive, multi-site, multi-disciplinary database offers a lot of promise. To address issues of data timeliness and evolving practice, a prospective and systematic data extraction approach has been developed for our group of registries that is performed quarterly. This works with the hospital EHRs and administrative datasets to identify all consecutive cases that meet the registries criteria. This ensures completeness of the cancer dataset as well as real-time data statistics that are linked with automated visual analytic software. Individual treating clinicians may also log onto the electronic web-based registry portals to update treatment plans in real-time.

Within comprehensive multi-site clinical registries, it is possible to examine the presence of variations in outcomes or practice across different treatment locations. Registry data can also identify associations of the putative quality indicator or metric with outcomes. For example, in a preliminary analysis of metastatic colorectal cancer, we found substantial variation in the rates of resection of liver metastases, which may correlate with survival data [10]. Further work is underway to understand the drivers of this variation and to address any deficiencies identified.

In tumour streams where there are multiple interventions, and biomarker-defined subsets, there is greater potential for variation and for compromised outcomes from poor quality care. Here, we are developing quality audit and reporting initiatives to define metrics across a range of disciplines. Appropriate biomarker testing and tumour staging which are essential to inform standard care are metrics that can be tracked. Other examples include performance of *RAS*, *RAF*, and MMR testing in metastatic colorectal cancer or pelvic MRI imaging in locally advanced rectal cancer. It is important to consider metrics that measure multi-disciplinary care to improve the overall pathway rather than focusing on a single craft group.

Registries can also improve our understanding of the evolving patterns of care and impact of new treatments beyond the clinical trial setting. This includes the uptake of new therapies and whether they are being adopted into routine clinical care, as well as the efficacy and toxicity of new treatment options in a real-world population. Thus, overcoming the limited external validity of trial data. Across multiple registries we have demonstrated similar if not better outcomes than those observed in the matching earlier clinical trial [11]. We postulate that this may reflect broader gains in multidisciplinary care over time, including an increasingly aggressive approach to treatment of oligometastatic disease, improved supportive care, and additional salvage therapies becoming available. This data also provides assurance to clinicians and patients that the survival outcomes being discussed, based on trial data, is generalisable and reproducible in a real-world setting, beyond the selective confines of a clinical trial.

### 5.2. Biomarker and Translational Research

Linking clinical data collected in a registry with matched biospecimen data opens up a broad range of opportunities to explore and evaluate putative biomarkers. Arguably, the independent validation of reported biomarkers is of the utmost importance; in 2019 Ruiz-Banobre et al. reviewed predictive markers in colorectal cancer, identifying 148 predictive biomarkers reported in the published literature, of which only two (1.4%) had been explored prospectively, and only 14 (9.6%) had been tested in an independent cohort [12].

The complex interaction between biomarkers or associations that may not be evident or captured in a clinical trial dataset is a particular opportunity for registry analyses which provide much larger datasets to contribute. For example, exploring the association and interactions between primary tumour side [13], *BRAF* V600E mutation, and MMR deficiency, all of which individually are prognostic and predictive factors [14], or novel biomarker associations between *RAS* mutations and lung metastases [15] or tumour stage impacting sites of recurrence [16] all demonstrate how the comprehensive registry data set provides information not captured in a clinical trial data set.

In the adjuvant setting, validating prognostic and predictive markers will enable identification and treatment of patients most likely to respond and avoidance of ineffective treatment for those unlikely to derive benefit. In a series of studies, we explored the utility of circulating tumour DNA (ctDNA) a promising marker of minimal residual disease. Initially these studies were observational, with a blood test being taken from patients receiving standard care for early stage [17] or metastatic disease [18]. Now a series of randomised studies are being pursued with a similar model, recording treatment and outcome data collected in the corresponding tumour registries. The first of these studies has recently been reported [19].

This approach is also being employed with other novel predictive markers. For example, *EGFR* ligand expression is a promising predictive marker for treatment with EGFR inhibitors in metastatic colorectal cancer [20]. Ideally prospective randomised trials are required to validate this, with biomarker driven treatment decisions confirming the true impact of biomarker informed care. Leveraging our translational activity, patient derived tumour organoid sensitivity testing is being pursued to guide personalised treatment selection, with matched clinical data collated in the existing registries.

Another emerging use of registry data is to identify clinical trial candidates, particularly where patient eligibility is biomarker-driven. A recent example is a database search to find colorectal cancer patients with a *KRAS* G12C mutation, noting that RAS testing data was already part of standard of care as a negative predictor of EGFR inhibitor efficacy. Identifying particular patient subsets also provides opportunity to understand the natural history of that biomarker subset, such as the impact of a *BRAF* V600E mutation in early stage [21] or advanced [22] colorectal cancer.

### 5.3. Registry-Based Clinical Trials

Randomised controlled trials (RCT) represent the gold standard for evidence generation, however this approach is typically expensive and often hindered by poor recruitment. Notably a recent study reported that only 0.1% of patients in the US national cancer database were enrolled on a clinical trial [23]. There is a widening gap between the number of critical clinical management questions that should be asked in an RCT and the trials that are addressing these, resulting in an ever-growing knowledge gap challenging the optimal use of available therapies. The need for a more economical and pragmatic approach to the execution of clinical trials has given rise to the concept of registry-based randomised controlled trials (rRCTs).

This new approach of rRCTs is gaining traction across medical disciplines, led by practice changing studies in cardiology that answered important questions by recruiting large cohorts at minimal cost. This new clinical trial paradigm, hypothesized to be “the next disruptive technology in clinical research” [24] provides an opportunity to address important clinical practice questions that are not being addressed via conventional clinical trials, pending the registry being fit for purpose [25]. rRCTs can build on existing registries as a platform for patient randomization, and, most importantly, for capture of patient, treatment and outcome data, leading to markedly reduced costs. Using registry data also means data collected prior to or after the study period is also accessible. Additional advantages of rRCTs would be enhanced generalizability where less stringent entry criteria are used, larger cohorts recruited, and substantially reduced costs.

Our initial efforts at registry-based trials [26] are exploring optimal treatment combinations or duration and pursuing head-to-head comparisons of existing standards of care not previously directly compared. More recent trials are focusing on outcomes in specific patient populations, such as the elderly, where a single arm prospective study can explore clinician decision making and treatment outcomes. Other concepts are being developed for palliative care, examining the use of medication at end of life, and consumer led trials. There is also potential to include patient interviews, such as in the REAL-PRO study [26], and patient reported outcome data in a registry trial.

## 6. Other Registry-Based Opportunities

Several other opportunities made possible through the collection of registry data include:

### 6.1. Health Economic Analyses

With the ever-increasing cost of cancer care we have an insurmountable challenge of providing all testing and all treatment options to every patient whilst using health care dollars as efficiently as possible. Understanding the cost–benefit ratio of a new treatment can be explored using registry data, which can provide estimates of the costs of patient management as these change over time [27]. For example, not treating a patient with a *KRAS* mutated metastatic colorectal cancer with an EGFR inhibitor saves many thousands of dollars of drug cost using a test that costs hundreds of dollars. Currently we are analysing the health economics of circulating tumour DNA as a biomarker of minimal residual disease in early stage colorectal cancer, utilising the registry data to model real-world resource consumption [28].

### 6.2. Patient Education

Another novel use of registry data is in providing patients with a personalised treatment summary (which they can use as a health record across a variety of settings) and a personalised treatment plan. The latter is most relevant to the adjuvant setting where a summary of treatment planned in the multi-disciplinary team meetings can be provided to the patient and the general practitioner, highlighting any planned therapy and surveillance.

### 6.3. Consumer Data Entry

The new frontier of registry data is integrating efforts with patient entered data, wearable monitoring technology such as Fitbit data and other evolving home monitoring health solutions. Of particular interest is combining registry data with patient diaries and electronic patient reported outcomes (ePROs) that capture the lived experience, including adverse events, which are not well captured in the medical record (and the registry). This record can also be used in real time when patients are undergoing clinical review and accurate recall of recent events can be challenging. To obtain the most value the consumer and clinical data should be maximally aligned, including providing opportunity for audit (through patients being able to access and review their registry data, and to question any potential errors if these were to occur).

### 6.4. Medicine Access Programs (MAP)

Prior to government funding of oncology agents, pharmaceutical companies may elect to allow early access to medications via a MAP, either self-funded by the patient or compassionately supported by the company. Despite the potential for comprehensive outcome data to be generated from patients who participate in MAPs, this data is traditionally not collected due to perceived ethical, data ownership, data security and data privacy issues. A registry developed alongside a MAP can yield valuable data on real-world efficacy and safety, and our group has demonstrated the feasibility of successful data collection in two key breast cancer registries, PERSIA and KARMA. These registries are independent of the pharmaceutical companies and governed by institutional research ethics, data and privacy rules.

### 6.5. Discrete Choice Experiments

Discrete choice experiments (DCEs) are novel surveys that can be readily incorporated into electronic registry platforms. DCEs ascertain real world user preferences through a series of hypothetical competing alternatives. In the realm of oncology, DCEs can be used to gain insights on the relative importance ascribed to treatment goals versus treatment side effects by patients and doctors. DCEs are regarded as a rich source of patient-centric data that can be used to promote shared decision making and enrich patient-orientated care. Beyond that, DCE results can also be used to guide pharmacoeconomic decisions and health policies (Table 2).

### 6.6. Artificial Intelligence

Increasingly registries need to incorporate automated extraction of structure data from EHRs if they wish to increase their efficiency. These capabilities can extract specific data fields from pathology systems, structured clinical reporting systems and administrative hospital data. However, it should be noted that quality checks for this method of data extraction is imperative. Automated data is not free from error. “Breaks” or “bugs” within the extraction process can lead to flawed data entry, so even automated data entry needs to be maintained and regularly monitored.

With advances in computational power, another application of comprehensive data sets captured in registries particularly when combined with matched biospecimens, is to utilise artificial intelligence, machine learning and ultimately deep learning to maximise understanding of prognostic and predictive clinicopathologic biomarkers across the cancer continuum. Such models have the potential to affect and personalise cancer management from screening, diagnosis, treatment to survivorship. They can also synthesize increasingly complex influences upon clinical decision making for busy clinicians to maximise efficiency and ensure contemporary evidence-based medicine supports their daily practice. This approach is currently being investigated through registries like the PURPLE Pancreatic Cancer Translational registry, which has been specifically designed with this digital mindset hoping to prepare and transform the humble clinical registry into a more powerful research tool for the future.

## 7. Cautionary Tales of Registry Data Use

While there is increasing enthusiasm for exploring the growing amount of “real world” data to address questions not covered in clinical trials, it is important that this is tempered with a balanced consideration of the quality of data collected, the limitations around the data being collected (such as progression free survival), and the potential for confounders. It is critical to define the types of research questions for which it is reasonable and appropriate to use registry data. Of note, any comparisons between treatment strategies in the real world, which are often the most interesting questions to ask when randomised trial data is not available, are also the most limited due to confounders. Missing data must be accounted for when analysing the data to avoid bias. Increasingly the use of synoptic reporting on standard reports and at multi-disciplinary meetings can help to reduce missing data extracted from EHRs. In-built registry data quality checks previously described help to limit missing data, completeness of variables and timeliness of the data collated, however a degree of missing data is inevitable in any registry and should be taken into consideration during the analysis process.

Measures to ensure reproducibility of results is another important consideration. This includes addressing data reproducibility, computational and statistical/model reproducibility, which becomes particularly important as we move into the realm of “big data” analyses, automated data extraction, integration of biological data with clinical data and artificial intelligence. Measures such as targeted data acquisition can improve data reproducibility [29], whilst detailed narrative descriptions and custom scripts and code automation are some measures that can improve computational reproducibility [30].

Any associations found using real-world registry data should be considered hypothesis generating. It is sobering to note that no relationship was found between comparisons made of different treatments when comparing these in routine care and the outcomes of a subsequent clinical trial that directly compared the two treatments [31].

Publication bias remains a challenge for registry-based research as there is no limit to the number of questions that can be asked. For any of these effectively retrospective series [32] there is a temptation to write up and publish the most interesting findings, and to not pursue others. These interesting findings can be due to confounders or chance observations, the likelihood of which is increased with selective entry criteria (patients or time periods) or endpoints, which can evolve over the time of the analysis. Notably for clinical trials the study aim, patient population and entry criteria are defined upfront in the protocol. In contrast, when examining registry data, the patient subset, the time period, and the specific question being asked can all potentially be manipulated to lead to more interesting results. Having a rigorously defined and approved plan before commencing any research project, as is our standard protocol, should reduce any temptation to “torture” the data in pursuit of a statistically significant result. We also expect research projects using our registries to publicly present and/or publish the findings, in order to minimise the risk of selective reporting, with the failure to do so limiting any further access to registry-based research.

## 8. Conclusions

Facilitating the collection of real-world multi-disciplinary data across multiple sites has the potential to improve our understanding of cancer biology, identify relevant prognostic and predictive biomarkers, facilitate translational research, and drive research efficiency. However, the promise contains much more. Collecting data on large numbers of patients across several domains can lead to impactful research, ensuring patients receive high quality care regardless of location, have their information needs met and supported, and experience better quality of survival.

## Figures and Tables

**Table 1:** Examples of data linkage and value obtained.

Local Database	Data Provided	Value
Electronic Health Records	Source data, structured clinical data reports, multidisciplinary meeting reports, imaging reports, pathology reports, treatment intent, start and end dates for treatment, treatment outcomes	Source data, and data quality
Pharmacy database	Therapy administered	Data quality
Familial cancer database	Testing and outcomes	Research [3]
Administration data	Patient admission and discharge dates, language spoken	Data audit and research [4]
**External database**		
Registry of births, deaths, marriages	Date of death and cause of death	Data quality
National death index	Date of death	Data quality
Pharmaceutical benefits service	Patient prescriptions	Data quality, additional medication data
Medical benefits service	Patient procedures	Data quality, additional data for audit and research
Cancer council	Date of diagnosis	Data quality [5]

**Table 2:** Example choice set in a discrete choice experiment comparing 2 hypothetical treatment options for kidney cancer patients.

	Treatment A	Treatment B
Effectiveness	Tumours shrink but persist and remain small for 2 years	Tumours could initially shrink completely but may grow in the future after a period of quiescence
Tiredness	70% feel extremely tired	10% feel extremely tired
Severe or life-threatening hormonal side effects	Risk of hormonal side effects is negligible	20% experience hormonal side effects that require treatment with steroids
Method of delivery	Tablets taken every day	Intravenous infusions given every 3 weeks

## Data Availability

Not applicable.
